# *Pseudomonas aeruginosa* Evolutionary Adaptation and Diversification in Cystic Fibrosis Chronic Lung Infections

**DOI:** 10.1016/j.tim.2016.01.008

**Published:** 2016-05

**Authors:** Craig Winstanley, Siobhan O’Brien, Michael A. Brockhurst

**Affiliations:** 1 Department of Clinical Infection, Microbiology and Immunology, Institute of Infection and Global Health, Ronald Ross Building, University of Liverpool, 8 West Derby Street, Liverpool, L69 7BE, UK; 2 Department of Biology, University of York, Wentworth Way, York, YO10 5DD, UK

**Keywords:** *Pseudomonas aeruginosa*, cystic fibrosis, evolution, adaptation, population biology

## Abstract

*Pseudomonas aeruginosa* populations undergo a characteristic evolutionary adaptation during chronic infection of the cystic fibrosis (CF) lung, including reduced production of virulence factors, transition to a biofilm-associated lifestyle, and evolution of high-level antibiotic resistance. Populations of *P. aeruginosa* in chronic CF lung infections typically exhibit high phenotypic diversity, including for clinically important traits such as antibiotic resistance and toxin production, and this diversity is dynamic over time, making accurate diagnosis and treatment challenging. Population genomics studies reveal extensive genetic diversity within patients, including for transmissible strains the coexistence of highly divergent lineages acquired by patient-to-patient transmission. The inherent spatial structure and spatial heterogeneity of selection in the CF lung appears to play a key role in driving *P. aeruginosa* diversification.

## Trends

During chronic lung infections of CF patients common genetic adaptations occur in *P. aeruginosa*, such as conversion to mucoidy, loss of virulence factors, and resistance to antibiotics.

Although pathoadaptive mutations in regulatory proteins are common, the actual regulators affected vary between populations.

*P. aeruginosa* populations in CF lungs exhibit high levels of phenotypic diversity.

Fine-scale population genomics approaches reveal that divergent sublineages can coexist, with evidence for regional isolation in the spatially structured and heterogeneous lung environment.

Experimental evolution is beginning to provide insights into the selective drivers of evolution in *P. aeruginosa* infection, including the role of social interactions.

## *Pseudomonas aeruginosa* Infection in Cystic Fibrosis

**Cystic fibrosis** (**CF**; see Glossary) is a debilitating, genetically inherited disease characterised by defects in a transport protein (the cystic fibrosis transmembrane regulator), resulting in sticky mucus, most notably in the respiratory tract [1]. CF patients are susceptible to chronic lung infections, the predominant cause of the morbidity and mortality associated with the disease. The most common pathogen in this respect is *Pseudomonas aeruginosa*, a highly versatile bacterium capable of causing a wide range of mostly opportunistic infections, as well as occupying a variety of environmental niches [2]. The ecological flexibility of *P. aeruginosa* can be attributed to its large genome (typically >6 Mb), which contains a particularly high proportion of regulatory genes, as well as a large number of genes involved in the catabolism, transport, and efflux of organic compounds 3, 4. In CF, there has been some progress with development of aggressive early eradication therapies, whereby treatment is initiated as soon as the pathogen is detected, which delays the onset of chronic infection [5]. However, once a chronic infection is established by *P. aeruginosa*, it is apparently impossible to eradicate.

During the course of chronic infection, CF patients produce samples (most commonly sputum) that are subjected to microbiological analysis for diagnostic purposes (identification of pathogens and antimicrobial susceptibility testing) and have proven to be a rich resource for researchers interested in analyses of the evolution of bacteria during chronic infection. As a discipline, microbiology has depended heavily on analysis of cultured organisms [6], and microbiologists are ingrained with the importance of obtaining single pure colonies. Hence, the study of bacterial pathogens during infection, including chronic CF infections, has relied heavily upon an assumption that bacterial populations at any given time are genetically uniform, at least at the level of ‘strains’, and that therefore it is justifiable to study and diagnose infections on the basis of single isolates. As a result, for many years researchers have assumed that it is possible to draw conclusions about the whole infecting bacterial population from the traits of single bacterial colonies. It is, however, increasingly clear that this assumption may not always be true.

In this review we discuss the phenotypic and genomic studies that have advanced our understanding of the adaptation and evolution of *P. aeruginosa* populations during chronic infections in the CF lung, highlighting the evidence demonstrating that infecting *P. aeruginosa* populations are highly diverse both genetically and phenotypically. We further discuss the causes and consequences of this diversity with respect to the underlying evolutionary processes and the clinical implications.

## *P. aeruginosa* Phenotypic Adaptations Commonly Associated with CF Infections

The CF lung is a heterogeneous, hostile, and stressful environment for invading bacteria, and *P. aeruginosa* populations must overcome these challenges to persist and survive. Postulated stressors in the CF lung include osmotic stress [7] due to the viscous mucus, oxidative [8] and nitrosative [9] stresses due to host responses, sublethal concentrations of antibiotics [10], and the presence of other microorganisms 11, 12. It has been recognised for many years that *P. aeruginosa* undergoes evolutionary changes in response to these selective forces during the chronic infection process. Phenotypic analysis of isolates show the emergence of mucoid colonies [13], caused by overproduction of the polysaccharide alginate, which is widely considered to be a marker for the transition to chronic infection. Alginate is one of three exopolysaccharides (along with Pel and Psl) that play important roles in the development and structural maintenance of a biofilm matrix that can offer *P. aeruginosa* protection from antibiotics and host responses [14]. Other adaptations include the accumulation of auxotrophic mutations in the amino acid-rich lung environment [15], loss of motility [16], and the emergence of hypermutators [17], which display elevated mutation rates due to defects in DNA repair mechanisms. Given that CF patients are subjected to prolonged and often intensive therapy with antibiotics [18], the evolution of antibiotic resistances is also a common adaptation [19].

**Figure 1:**
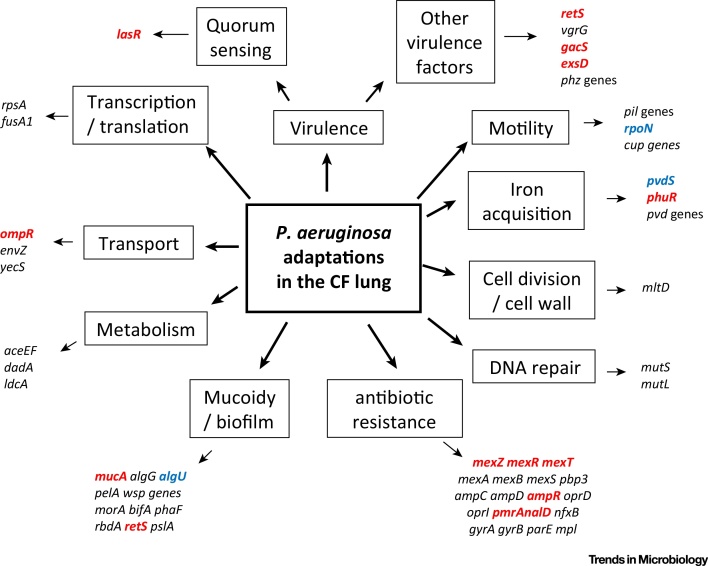
Pathoadaptive Mutations in *Pseudomonas aeruginosa*. Genes encoding regulatory proteins are highlighted in red. Genes encoding sigma factors are highlighted in blue.

A recurring theme in the phenotypic analysis of CF isolates is the tendency for *P. aeruginosa* to become defective in terms of some of its key virulence factors (Box 1), such as type III secretion and the **quorum sensing (QS)** system (Figure 1). In CF, the accumulation of mutations in the gene encoding the key QS regulator, LasR, causing loss of QS regulation, is especially common [20]. Other mutations often found associated with CF isolates of *P. aeruginosa* include mutations in *gacS* and *retS*, genes implicated in the switch between acute and chronic virulence (Box 1, Figure 1). Other regulators, such as AmpR [21], have also been implicated in this switch to chronicity, and it seems likely that a number of global regulatory systems may actually be involved. The accumulation of virulence factor mutations has been interpreted as *P. aeruginosa* adapting to lose its acute virulence during chronic infections [22]. However, it is clear that many virulence factors (for example, QS-regulated factors and QS signal molecules) can still be detected in patient sputum samples during chronic infections [23].

## Genomic Analysis of Adaptation Using Sequential Isolates

The advent of affordable whole-genome sequencing technologies triggered considerable interest in using genomics to define the genetic basis of adaptations that occur during infections in the CF lung environment. In particular, there have been a number of studies reporting comparisons of clonally related longitudinal isolates, mostly contrasting the mutated genes in isolates from early and late in the infection process 24, 25, 26, 27, 28, 29. These studies have revealed common mutations falling into various functional categories such as virulence (including QS and mucoidy), motility, transport, antibiotic resistance, iron acquisition, DNA replication or repair, transcription/translation, cell division or metabolism. In particular, mutations in genes encoding key global regulators are common (for example, *lasR*, *rpoN*, *mucA*, *mexT*, *retS*, *exsD*, and *ampR*; see Figure 1). Together, the suite of traits affected by these mutations have been termed ‘pathoadaptive’ traits.

An analysis of an extensive retrospective collection of isolates, many of which represented a transmissible lineage (DK2), demonstrated that after initial transmission, sublineages evolved independently in patients, accumulating pathoadaptive mutations [25]. It was further demonstrated that hypermutator lineages can coexist with nonhypermutators, developing distinct evolutionary pathways [30]. Expanding this work to a study of 474 longitudinal isolates from 34 CF children and young adults, representing 36 different lineages of *P. aeruginosa*, the same group were able to show parallel evolution at 52 genes [31], suggesting common adaptations and constraints during the process of adaptation.

Each of these studies suggests evidence for adaptive evolution, with selection for mutations that are beneficial in the CF lung environment. For example, there is evidence for adaptation towards iron acquisition from haemoglobin repeatably and independently across multiple patients [32]. Notably, mutations of the complex *P. aeruginosa* regulatory and metabolic networks in the lung environment are likely to extensively modify gene expression levels and alter metabolic fluxes. Mutations occuring early in the infection, and therefore presumably the most beneficial, are located in global regulatory network control hubs [33], with other mutations occurring later and leading to fine-tuning [33]. However, it is also notable that the pathoadaptive mutations are not consistent between studies, suggesting the existence of multiple evolutionary trajectories to pathoadaptation [34]. Observations in CF suggest that this may be a result of the inherent complexity of *P. aeruginosa* regulatory networks [35]: similarly beneficial effects can result from different mutations, or combinations of mutations in regulators, leading to changes in multiple processes. As a result, different mutations may converge upon similar phenotypes and levels of increased fitness. Likewise, there may be epistatic interactions among mutations such that particular combinations of mutations are required, making the evolutionary trajectory within a given patient highly contingent upon which early mutations arise and reach fixation [34].

The tendency for *P. aeruginosa* to acquire loss-of-function mutations during adaptation to the CF lung could suggest that it is travelling towards an evolutionary ‘dead-end’. However, the existence of transmissible strains, such as the DK2 lineage and the **Liverpool epidemic strain** (LES) 36, 37, argues that this is not always the case. Anecdotally, the LES is most likely to infect patients already infected with another *P. aeruginosa* strain. It is conceivable, therefore, that transmissible strains have acquired mutations that not only favour transmission but also enhance competitive ability in the lung.

## What Are the Drivers of Pathoadaptation?

Parallel evolution of particular traits or genes independently in multiple patients is strongly suggestive of positive selection at these loci, leading to the identification of the suite of pathoadaptive traits. Thus, we now have detailed phenotypic and genetic descriptions of how natural selection targets *P. aeruginosa* populations in the CF lung, but we still lack a full understanding of why these particular traits and genes are experiencing selection. This is in part due to the fact that the CF host environment is highly complex, and in part a reflection of our incomplete understanding of the physiology of the bacteria. Perhaps the clearest case where the selective force can be linked to the evolutionary response is for antibiotic resistance evolution: for example, in a recent genomics study of *P. aeruginosa* adapting to the CF lung, the fitness of particular alleles at the penicillin-binding protein 3 could be linked to the use of particular antibiotics [38]. Similarly, in *in vitro* evolution experiments, stereotypical resistance mutations become enriched under antibiotic selection, clearly establishing causality [39]. Other traits are less easily associated to particular selective causes. For example, it has been suggested that the loss of virulence-associated and motility traits is a response to immune selection [16]; however, there have been few direct tests of this hypothesis. Indeed, a recent study using nematode hosts showed no effect of host immunity on the trajectory of *P. aeruginosa* adaptation: virulence traits were lost with and without immune selection [40]. Similarly, adaptation to the CF-like conditions of artificial sputum medium selects for mutations that cause a switch to an immotile biofilm lifestyle [39], suggesting that the sputum environment itself is sufficient to select against motility. Moreover, growth of *P. aeruginosa* in flow cells selects for mutations causing mucoidy and loss of pilus-dependent motility, suggesting that simply dwelling in a biofilm is sufficient to cause the evolution of these characteristic CF-associated phenotypes [41]. Other common adaptations include changes in metabolism, DNA repair, and iron acquisition (Figure 1). However, there is a clear need for careful experiments testing evolutionary hypotheses about the drivers of selection within the host which disentangle this complex multifaceted environment. Box 2 outlines our current knowledge with respect to adaptations driven by social interactions, demonstrating how experimental evolution can help us to both generate and test hypotheses.

## Complexity of *P. aeruginosa* Populations in the CF Lung

**Figure 2:**
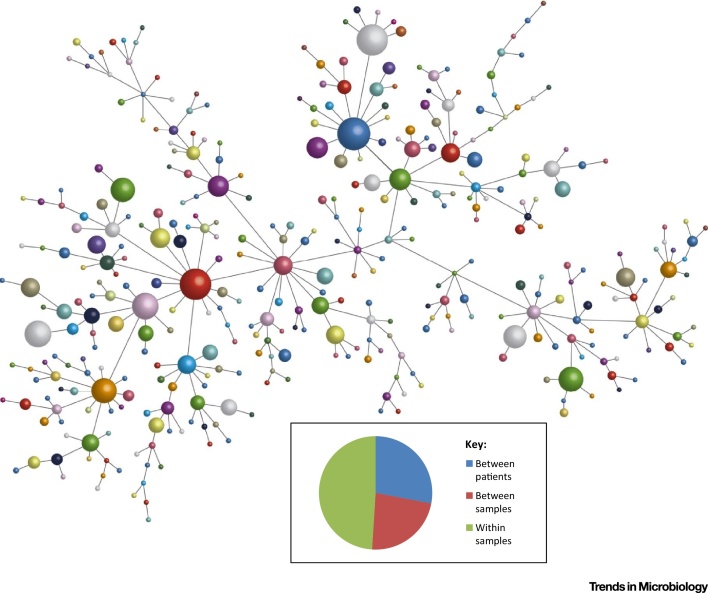
Key Figure: Phenotypic Heterogeneity within *Pseudomonas aeruginosa* Populations in Cystic Fibrosis (CF)

**Figure 3:**
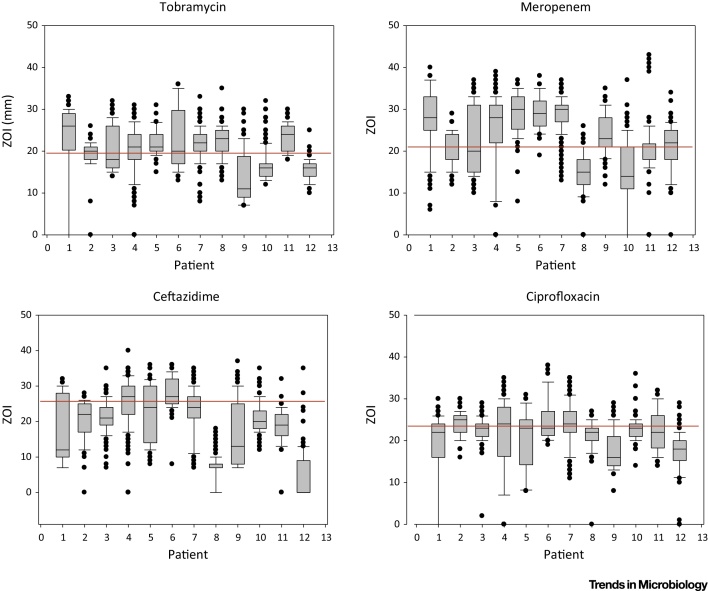
Variations in the Antimicrobial Susceptibilities of *Pseudomonas aeruginosa* within Individual Patients. The figure summarises, for four antibiotics, the spread of zone of inhibition data (ZOI) in mm for multiple isolates taken from 13 patients infected with the Liverpool epidemic strain (LES). For each patient, a minimum of 80 isolates was analysed, taken at multiple sampling points (40 isolates per sample point). The red line indicates the recognised cut-off points as defined by the British Society for Antimicrobial Susceptibility [84]. Note the tendency for isolates from the same patient to occur both above (susceptible) and below (resistant) the red line. Data adapted from two studies 43, 46.

It has been known anecdotally for many years that *P. aeruginosa* populations in CF can be diverse in terms of phenotypes such as colony morphology (e.g., coexistence of mucoid and nonmucoid colonies). Recent detailed analyses have revealed that there is extensive phenotypic heterogeneity within populations of *P. aeruginosa* in the CF lung beyond their colony morphology 42, 43, 44, 45, 46, 47, 48, 49 (Figure 2, Key Figure). Importantly, *P. aeruginosa* populations exhibit within-population diversity in many of the phenotypes observed to be altered during evolutionary adaptation, including motility, virulence factor production, siderophore production, antibiotic resistance, auxotrophy, and hypermutability. Hence, although some members of the population have acquired mutations affecting these phenotypes, these mutants coexist in patients alongside other genotypes that have not (Figure 3). The most practical clinically relevant consequence of this phenotypic diversity is in relation to antimicrobial susceptibility testing in diagnostic laboratories, which is typically carried out on either single isolates, or two colonies acting as representatives of mucoid and nonmucoid colony morphotypes. However, there is almost always considerable diversity in the antimicrobial susceptibilities within the population isolated from an individual sputum sample (and within colony morphotypes) 43, 45 (Figure 3). Hence, it is perhaps not surprising that therapy based on antimicrobial testing is a poor predictor of clinical outcome 50, 51.

**Figure 4:**
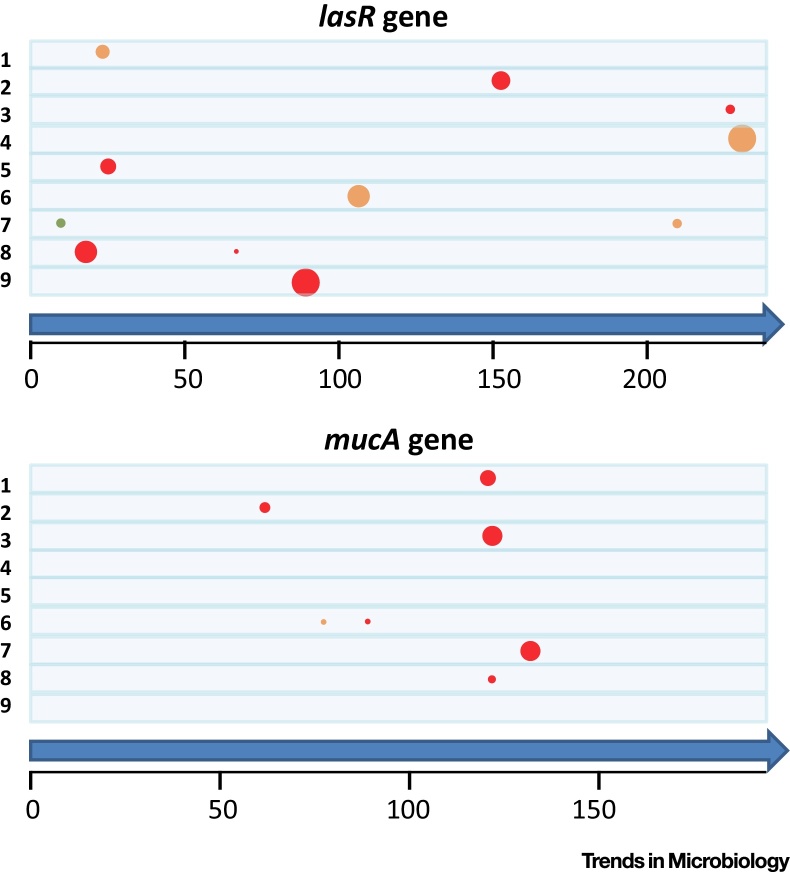
Within-patient Variations in the Prevalence and Location within a Gene of Common Pathoadaptive Mutations. Based on sets of 40 isolates from a single sputum sample, for each of nine patients infected with the Liverpool epidemic strain, the prevalence and location of mutations is indicated for the *lasR* and *mucA* genes (with location relative to amino acid position on the predicted protein sequence indicated on the scale). Each patient is represented by the space between lines. Each circle represents the location of a mutation that is either severe (red; e.g., a frame-shift), a nonsynonymous single nucleotide polymorphism (orange; e.g., a single amino acid change), or a change that would not impact on the protein sequence (green). The size of the circle reflects the relative abundance in each set of 40 isolates (e.g., the severe *lasR* mutation in patient 9 was present in all 40 isolates tested). Analysis of data from a published study [53].

This closer scrutiny of *P. aeruginosa* within-population diversity has been extended to genomic analysis, with reports that contemporary within-patient genomic diversity between isolates can be comparable to the variation reported between sequential isolates [52]. Further studies have built upon this by analysing larger numbers of isolates per sample, to characterise the diversity present within populations 44, 53. By sequencing *P. aeruginosa* populations from a group of patients infected with the same strain (the LES), it was possible to demonstrate the coexistence of divergent sublineages within individual patients, strongly suggesting the likelihood of ongoing transmission between patients [53]. Data from this study also demonstrated that, although mutations in some genes are common, they are neither always present in all patients, nor carried by all members of the *P. aeruginosa* population within individual patients (Figure 4). Further evidence for the merits of studying fine-scale evolutionary dynamics was provided in another recent paper, which reported deep analysis of 12 sputum samples isolated from one patient over the course of a year [38], with the evolutionary emergence of two clonal sublineages within the patient.

This observed diversification is consistent with the idea that genetically diverged isolates coexist and interact within an ecologically cohesive population in the lung. However, there is likely to be a role for spatial structure and environmental heterogeneity within the lung environment in the origin and maintenance of the observed genetic diversity. Different regions of lung tissue are likely to vary along a range of environmental axes, including variations in mixtures of nutrients, concentrations of penetrating antibiotics, other microorganisms, or factors such as oxygen availability, potentially leading to spatially variable selection for different ecotypes. At a macro-scale this is supported by the coexistence of distinct sublineages associated with different parts of the airway in patients (paranasal sinus vs lung) [54]. Moreover, in a recent landmark paper, different regions of explanted lungs from chronically infected CF patients were analysed to study the variations in clonally related isolates in great detail [55]. The *P. aeruginosa* isolates occupying different regions of the lung had evolved independently, and they differed in phenotypic characteristics such as nutritional requirements, antibiotic resistance, and virulence. The study, however, reported that there was limited intermixing between these separate communities, suggesting that regional isolation mediated by spatial structure promotes both the origin and the maintenance of this diversity [55]. It is important to note that spatial divergence of this kind could arise simply due to random drift rather than any adaptive divergence.

Therefore, upon current evidence, it appears that the observed diversity in CF lungs is the result of both adaptive (spatially heterogeneous selection driving the evolution of different ecotypes in different regions of the lung) and nonadaptive (genetic drift due to spatial structure combined with limited mixing) processes that promote the origin and coexistence of genotypes. Although recombination can also play a role in the diversification process 44, 52, 53, 56, there is disagreement about the extent of its contribution. One study suggested that recombination is a key driver of genomic and phenotypic diversity [44]. However, refined analysis of these data [57], and the levels reported in other studies, suggest that recombination rates are low. Although experimental tests are limited, it has been shown in several studies that genetic diversity readily evolves in spatially structured biofilm populations 41, 58, 59, 60. Moreover, several studies have demonstrated a role for stressors likely to occur in the CF lung in selecting for the evolution of diversity in *P. aeruginosa* biofilms (oxidative stress [59]) and in populations growing in a CF-like artificial sputum medium environment (subinhibitory concentrations of antibiotics [61]). The complexity of the system is emphasised by the fact that diversity can occur both within a biofilm population in a ‘microcompartment’ and in the wider CF airway environment, where physically separate biofilm populations can be exposed to different environmental conditions, including variable antibiotic concentrations [62]. Furthermore, as the lung tissue deteriorates over time during CF disease progression, it is likely that the selective forces operating on the pathogen population vary, such that mutations beneficial earlier in the infection may be less favoured later.

The effects of the many selective forces likely to operate in the lung remain to be experimentally tested. These include the other members of the complex multispecies microbial communities (the microbiome) [63], the host immune system, and phage (which can occur at high densities in CF patient samples [64]). Experimental evolution approaches are likely to be a powerful tool for shedding light on these issues.

## Concluding Remarks

Driven by the advent of affordable high-throughput genome sequencing, there has been rapid progress in our understanding of how *P. aeruginosa* adapts and evolves in the context of chronic CF lung infections. More recently, this has extended to fine-scale analysis of evolutionary dynamics within infecting populations, revealing high levels of coexisting genetic and phenotypic diversity, including at clinically important traits. The clinical consequences are not fully understood, though, given the extensive phenotypic diversity, there are clear implications for false diagnoses based upon antimicrobial susceptibility testing using single/pairs of isolates. Given the limited efficacy of current antibiotics in these chronically infected patients, it is important that we improve our understanding of the evolution of bacterial populations during chronic infections in order to design better strategies for clinical intervention (see Outstanding Questions). For example, the issue of whether the loss of social traits in *P. aeruginosa* populations is a result of disuse or cheating has relevance that extends beyond the field of evolutionary biology *per se.* If cheating can indeed accelerate the loss of virulence-associated metabolites 65, 66, meddling in the social lives of microbes could present a novel strategy for the development of virulence-attenuating therapeutics, as well as controlling the spread of infectious diseases 67, 68. In particular, there has been particular emphasis on developing novel antivirulence therapeutics, especially strategies aimed at inhibition of the *P. aeruginosa* QS system 69, 70.

Given the tools at our disposal, we are now well placed to undertake a detailed characterisation of the structure and dynamics of bacterial populations during infections, and it is likely that a huge amount of data will be published over the coming years. However, it is also important that we accelerate our understanding of the fundamental biology underlying the complexity of pathogen physiology and evolution. One key approach to bridging the gap between *in vivo* observational data and an understanding of the biological mechanisms is to use data from clinical samples to generate hypotheses that can be tested subsequently using experimental evolution. By using an iterative cycle of these kinds of approaches we have the potential to unpick the, often daunting, complexity of real microbial populations during the infection process.

Outstanding Questions What are the clinical consequences of evolution and high, dynamic diversity in *P. aeruginosa* populations for CF patients?

Can the complex within-host selective environment be unravelled in order to understand why particular *P. aeruginosa* traits experience selection in infections and how diversity is maintained?

Can the evolutionary trajectory of *P. aeruginosa* in CF lung infections be manipulated to improve patient outcomes?

## Box 1 Pseudomonas aeruginosa Virulence Factors

*P. aeruginosa* virulence has largely been defined in terms of acute infections, for which models are readily available and loss of function mutations can be easily demonstrated 71, 72, 73. The expression of virulence genes in *P. aeruginosa* is controlled by extremely complex, interweaving regulatory circuits [35] and multiple signalling systems [74]. For an opportunistic pathogen, *P. aeruginosa* produces an impressive array of particularly secreted virulence factors, utilising its type III secretion system to secrete various exotoxins (ExoS, ExoU, ExoT, and ExoY) [75], and quorum sensing (QS) systems (cell-density-dependent regulation) to control numerous important secreted virulence factors 23, 76, including secreted pyocyanin, elastase, cyanide, and rhamnolipid. The secretion of toxins, coupled with phenotypes such as motility (swimming, swarming, and twitching), is considered to be important for acute infections. In contrast, apart from biofilm formation, virulence factors that are important in chronic infections are less well understood. It has been proposed that the **GAC system** network (incorporating the two-component regulatory system GacA/GacS, two other sensor kinases RetS/LadS, the small regulatory protein RsmA and the small RNAs RsmZ/RsmY) controls the reversible transition from acute to chronic infections [74]. This is based on a switch between acute virulence factors (swarming motility, lipase, rhamnolipids and type III secretion) and chronic virulence factors, such as biofilm formation, but also type VI secretion, and QS-regulated factors more traditionally thought of as involved in acute infections, namely pyocyanin and hydrogen cyanide. Although a role in acute infections has been demonstrated in animal models [77], because of the limitations of the available animal models of chronic infection, the role of the GAC system in chronic infection is less clear. However, an improved natural inhalation model that does not require the implantation of agar beads, and during which the bacteria adapt and exhibit features of chronic infections, has been developed [78]. This offers considerable potential to better characterise key chronic infection virulence factors.

## Box 2 Adaptation in the Context of Social Evolution

The transition from acute to chronic infection can radically alter costs and benefits associated with the secretion of extracellular metabolites. Some metabolites are costly to produce when not needed, which may explain the redundancy of virulence-associated genes observed during cystic fibrosis (CF) infections. However, secreted metabolites are often costly to produce, so even when they are crucial for growth their loss might be selectively advantageous – providing there remain enough producing individuals to compensate for this small reduction in metabolite concentration. Hence, nonproducers (‘cheats’) can still reap the benefits of metabolites produced by others (‘cooperators’), while paying little or no cost [79]. For instance, individuals that produce the costly iron-scavenging siderophore pyoverdine can be exploited by pyoverdine-negative mutants, who benefit from increased iron-acquisition without paying a cost. Experimental evolution has revealed that in spite of cheats exhibiting reduced relative fitness in an iron-limited medium, they can spread through a population of cooperators, over a matter of days 80, 81. A recent significant paper shows that social selection may be similarly resulting in pyoverdine mutants in the CF lung [82]. Loss-of-function mutations occur in pyoverdine synthesis genes, but not in the receptor gene (at least as long as producers and nonproducers coexist together), indicating that the loss of pyoverdine *in vivo* is not driven by disuse, but by loss-due-to-cheating. Cheats can only exploit producers as long as they maintain a functional receptor. If loss were a result of disuse, mutations would be predicted to accumulate in both receptor and synthesis genes. However, with the notable exception of pyoverdine, we know very little in practice about the role of social evolution in shaping trait loss in the context of the CF lung. It could be tentatively suggested that virulence factors and secreted toxins may benefit bacteria other than the producer, and hence may also be vulnerable to nonproducing cheats. If this is the case, the dominance of the ‘loss-due-to-disuse’ framework for explaining loss of secreted traits in CF lung infections may be open to uncertainty.

## Glossary

Cystic fibrosis (CF): the most common life-threatening genetically inherited disorder among Caucasians.
GAC system: the global activator of antibiotic and cyanide synthesis system in *P. aeruginosa* that incorporates a two-component regulatory system (GacS/GacA) and is thought to play a role in the switch from acute infection to chronic infection.
Liverpool epidemic strain (LES): the most common clone of *P. aeruginosa* among UK CF patients.
Quorum sensing (QS): a cell-density-dependent regulatory system controlling the expression of multiple genes.
